# Editorial: Amaranthus: Naturally Stress-Resistant Resources for Improved Agriculture and Human Health

**DOI:** 10.3389/fpls.2021.726875

**Published:** 2021-07-15

**Authors:** Chance W. Riggins, Ana Paulina Barba de la Rosa, Matthew W. Blair, Eduardo Espitia-Rangel

**Affiliations:** ^1^Department of Crop Sciences, University of Illinois at Urbana-Champaign, Urbana, IL, United States; ^2^Instituto Potosino de Investigación Científica y Tecnológica (IPICYT), San Luis Potosí, Mexico; ^3^Department of Agricultural and Environmental Sciences, Tennessee State University, Nashville, TN, United States; ^4^Campo Experimental Valle de México, Instituto Nacional de Investigaciones Forestales, Agrícolas y Pecuarias (INIFAP), Texcoco, Mexico

**Keywords:** amaranthus, orphan crops, salinity stress, defoliation, hypertension, whole genome sequencing

Adapting agriculture to global climate change, shifting patterns in food consumption, fulfilling the priorities of national and international programs, and instability in agricultural research funding trends will all be crucial challenges in the coming decades. Over the past 50 years, food supplies worldwide have trended toward increased homogeneity and interdependence on a limited number of major crop commodities (Khoury et al., 2014). This narrowing of crop diversity has substantial implications for future global food security and, perhaps more importantly, for human health with food quality as much a concern as quantity (Dwivedi et al., 2013). Addressing micronutrient deficiencies through biofortification has been a priority for major cereal and legume crops (Blair, 2013), but could equally be important in the sense of dietary diversification (Miller and Welch, 2013) and utilization of high mineral and high essential amino acid pseudocereals, such as grain amaranths, or vitamin-packed leaf vegetable species of the *Amaranthus* genus.

Current crop production patterns are not guaranteed given climate change predictions (Lobell et al., 2008; Wheeler and von Braun, 2013), mounting pressures to reduce environmental impacts of agriculture on soil health and water resources (Foley et al., 2005; FAO and ITPS, 2015), and other stressors that impact agricultural productivity (Borrell et al., 2020; Halsch et al., 2021). To keep pace with these pressures while maintaining yields, modern cultivars require continual development using genetic resources that must be conserved, evaluated for utility, and ultimately made accessible to breeders and farmers (Gepts, 2006). Wild relatives of crops are valuable sources of diversity and possessing useful traits to improve modern cultivars, but these plants are often underrepresented in germplasm repositories and under-prioritized for conservation (Khoury et al., 2019).

Orphan crops—i.e., cultivated plants that are under-utilized by comparison to major crops—are another diverse resource to foster resiliency in our food production systems, but suffer from similar challenges facing crop wild relatives due to research gaps and low prioritization for investment and conservation (Naylor et al., 2004). Thus, there are urgent needs for more research and conservation initiatives devoted to these resources to safeguard agricultural biodiversity, improve nutrition, and security of our global food supply. These issues provided the impetus for this Research Topic which focuses on *Amaranthus* species as promising resilient crops for the future.

Despite their wide history of use in many parts of the world, grain and vegetable amaranths are still considered orphan crops (FAO, 2018). The genus contains several domesticated or semi-domesticated species, many local landraces, little-studied wild species, and relevant weedy species, which together represent a broad spectrum of untapped genetic diversity (Shukla et al., 2017). Amaranths are increasing in popularity due to their favorable agronomic traits, including rapid growth rates, C4 photosynthesis, dual-uses for vegetable (leaf) and grain (seed) production, and tolerance to heat, drought, and salinity stress (Achigan-Dako et al., 2014; Hoidal et al., 2019). Amaranths are extraordinarily nutritious, and seeds produce several bioactive compounds of relevance for lowering the risk of chronic diseases (Velarde-Salcedo et al., 2019). These attributes make amaranths appealing models for basic and applied research and ripe for development as multidimensional crops—themes of which are explored in this Research Topic.

New findings have shed light on amaranth biology, ecology, and human-health benefits (Das, 2016; Velarde-Salcedo et al., 2019), yet questions remain toward improving our understanding of agronomic and adaptive traits in the genus. Three papers featured in this Research Topic contribute new knowledge to stress tolerance mechanisms in three distinct amaranth species grown as different crops in different parts of the world. Two of these papers by Estrada et al. and Sarker and Oba focus on salinity tolerance. Soil salinity poses a serious threat to agricultural production and increasingly contributes to land degradation worldwide (Panta et al., 2014; Cheeseman, 2015). Improving crops for salinity tolerance through conventional breeding has proven difficult due to the complexity of this trait, which has created interest in halophytic orphan crops like amaranth and quinoa in recent years. As highlighted by Estrada et al., the Amaranthaceae family comprises the highest proportion of halophytic species, and both amaranth and quinoa possess high genetic variability and adaptability to grow under salinity stress. Yet, few comparative studies with both species have been conducted. Their findings show different anatomical, physiologic, and molecular responses in *A. caudatus* and quinoa accessions under salt stress while still maintaining seed yield, which points to developing strategies for improving salt tolerance in these species.

In comparison, Sarker and Oba add to knowledge of salinity tolerance and the role of antioxidant mechanisms for *A. tricolor*, a popular inexpensive and nutritious leafy vegetable consumed throughout Southeast Asia where salt-affected land is particularly problematic. Stress caused by defoliation impacts growth and production in different ways depending on developmental stage and intensity level. Cisneros-Hernandez et al. investigate defoliation tolerance in *A. cruentus*, one of the most widely planted grain amaranth species, at different developmental stages and consequences on panicle emergence and flowering. These authors identified differences in carbon metabolism and allocation patterns as influenced by class II trehalose-phosphate synthase (TPS) genes. Trehalose-6-phosphate has emerged as an important signaling compound for plant growth and is known to help some plants withstand long periods of desiccation (Paul et al., 2018). Novel insights from these studies will undoubtedly inspire future work on the metabolites and biochemical pathways linked to stress response in *Amaranthus* and other crop species.

Although genomic resources for several cultivated and weedy *Amaranthus* species are available (Clouse et al., 2016; Gonçalves-Dias and Stetter, 2020; Montgomery et al., 2020), Deb et al. have contributed a valuable new reference genome of a superior local landrace variety of *A. hypochondriacus* from India. The significance of this study is not only a reference genome more suitable for breeding South Asian varieties of *A. hypochondriacus*, but also in demonstrating a methodology that could be applied to other local landraces and orphan crops in developing countries. This study is a welcome addition and complements previous efforts by Wu and Blair (2017) and Stetter and Schmid (2017) in genotyping by sequencing for SNP discovery.

Many studies have explored the chemical composition of amaranths revealing an exceptional nutritional balance compared to staple crop species. In this Research Topic, Nardo et al. reviews progress in this area over the last decade focusing on amaranth's promising role as a functional food with antihypertensive properties. Amaranth contains several bioactive compounds, including encrypted peptides with inhibitory functions against enzymes of the Renin-Angiotensin-Aldosterone System, which are targets of commonly prescribed pharmaceutical drugs for controlling hypertension. These bioactive compounds show promise for formulation of novel functional foods, but the authors also address the challenges related to demonstrating biological activity in complex food matrices.

Altogether, the papers presented in this Research Topic highlight the multidimensional benefits of both grain and vegetable amaranths and will facilitate interdisciplinary research for more climate-resilient varieties of orphan crops for sustainable food production across the world and improved human health among vegetarians and low-income consumers dependent on grain based diets.

## Author Contributions

All authors listed have made a substantial, direct and intellectual contribution to the work, and approved it for publication.

## Conflict of Interest

The authors declare that the research was conducted in the absence of any commercial or financial relationships that could be construed as a potential conflict of interest.
